# Editorial: Widely used and novel approaches of the measurement of arterial stiffness and central hemodynamic parameters: Is there a consensus on the horizon?

**DOI:** 10.3389/fphys.2023.1167257

**Published:** 2023-02-28

**Authors:** János Nemcsik, Christopher Clemens Mayer, Andrea Guala, Dimitrios Terentes-Printzios, Bart Spronck

**Affiliations:** ^1^ Department of Family Medicine, Faculty of Medicine, Semmelweis University, Budapest, Hungary; ^2^ Center for Health & Bioresources, AIT Austrian Institute of Technology, Vienna, Austria; ^3^ Vall d'Hebron Research Institute (VHIR), Barcelona, Spain; ^4^ CIBER-CV, Instituto de Salud Carlos III, Madrid, Spain; ^5^ 1st Cardiology Department, Hippokration Hospital, National and Kapodistrian University of Athens, Athens, Greece; ^6^ Department of Biomedical Engineering, CARIM School for Cardiovascular Diseases, Maastricht University, Maastricht, Netherlands; ^7^ Macquarie Medical School, Faculty of Medicine, Health and Human Sciences, Macquarie University, Sydney, NSW, Australia

**Keywords:** arterial stiffness, central blood pressure, pulse wave reflection, cardiovascular risk, modeling

The evaluation of vascular biomarkers can help to identify patients with high cardiovascular risk providing opportunity for early preventive interventions to avoid clinical endpoints like cardiovascular morbidity and mortality. Recently a consensus paper was published on the clinical use of pulse wave velocity (PWV) (Park et al., 2022) suggesting that in the near future the importance of PWV measurements in everyday clinical practice will increase (Vlachopoulos et al., 2015).

This Research Topic provides nine articles (one review paper and eight original research papers) that deepen the knowledge in the field. The wide scope of these articles includes the role of circulating biomarkers in vascular ageing, *ex vivo* animal experiments, technical developments in the measurement of aortic stiffness, reference values of wave reflection parameters as measured in humans, acute effects of vasoactive drugs on arterial stiffness metrics, and a meta-analysis of the effects of antihypertensive medications on central hemodynamic characteristics.

The review paper by Gopcevic et al. gives an overview of circulating biomarkers related to vascular ageing including oxidative stress-based, inflammation-based, cell matrix-based and epigenetic-based biomarkers, the process of DNA methylation, and the importance of telomere length. This paper is a report from an international scientific collaboration on vascular ageing, called VascAgeNet (Climie et al., 2020a; Climie et al., 2020b).

De Moudt et al. demonstrated in isolated mouse aortic segments that both the extracellular matrix and vascular smooth muscle cells contribute to pressure-dependent stiffness hysteresis. Stiffness hysteresis phenomena were contraction-, age-, and extracellular matrix-dependent, which suggests that vascular smooth muscle cells in interaction with the extracellular matrix contribute to the pressure-dependent pulse dampening in the aorta. These results warrant future studies in understanding the role of contractile cells of the aorta in arterial stiffening.

In the same *ex vivo* setup, Neutel et al. demonstrated that pulse pressure not only modulates the general stiffness of the tissue but also affects the physiological function of vascular smooth muscle cells. At lower pulse pressure the aortic segment showed a larger response to contractile stimuli strongly modulating stiffness, while at a higher pulse pressure, a smaller contraction-induced stiffness modulation was observed. This study contributes to the understanding of blood vessel biomechanics and could potentially yield new therapeutic insights.

Malik et al. provide an easier and less operator-dependent method based on ultrasound images to evaluate carotid artery mechanical properties. In 500 subjects, the single M-line ultrasound approach using the middle M-line was as accurate as the multiple M-line approach using 17 M-lines. Diameter, intima-media thickness, and Young’s elastic modulus were not statistically significantly different when using one vs. 17 M-lines. Differences in distension and distensibility coefficient, though statistically significant, were of a clinically irrelevant magnitude.

Laser Doppler velocimetry enables contactless measurement of carotid-femoral pulse wave velocity, however, the developed CARDIS device requires now additional quality assessment which hampers its clinical usefulness. Seoni et al. explored two possible methods (template matching and matrix profile) for quality assessment to enable real-time feedback on signal quality in future versions of the device. The authors benchmarked these methods using visual scoring as a reference. Both methods were found to be suitable for the automated assessment of the signal quality of acceleration data measured from the skin at the neck and groin.

In the paper of Giudici et al., the authors used mechanistic correction of carotid PWV to distinguish acute and chronic effects of blood pressure on local arterial stiffness. They found that when comparing healthy individuals with patients with stage I and II hypertension, after mechanistic blood pressure correction, the difference in carotid PWV between the two groups was reduced by 68% and became statistically non-significant. These results suggest that moderate hypertension in middle-aged subjects has minor impact on common carotid artery stiffness independently of the acute effect of blood pressure. Further clinical investigations are required to evaluate the clinical utility of this method.

In the article of Fortier et al., the authors demonstrated the acute effect of nitroglycerine on the vasculature using an arterial stiffness gradient (the ratio between aortic PWV and muscular PWV). Besides preserved aortic stiffness, nitroglycerine acutely decreases muscular stiffness and wave reflection enabling higher pulsatility to reach the microcirculation. This effect could be harmful in the longer term, especially for high flow/low resistance organs like the kidneys and brain. The arterial stiffness gradient can potentially have stronger prognostic value than aortic (carotid-femoral) PWV *per se*, and its changes due to intervention using vasoactive agents (like antihypertensive drugs) could also bear clinical importance.

Zocalo and Bia provide very important age, height, and sex-specific reference intervals for pulse wave analysis/wave separation analysis-derived parameters like augmentation pressure, augmentation index, forward and backward components of aortic blood pressure, reflection magnitude, and reflection index. The age range of the reference population (*n* = 1,688) is between 2 and 84 years. These data form an important step in the future clinical utility of these parameters.

Cheng et al. performed an individual data meta-analysis on the effects of older and newer antihypertensive agents on central hemodynamic parameters. They found a more pronounced decrease in central systolic blood pressure, augmentation index, and augmentation pressure with newer antihypertensive agents (angiotensin converting enzyme inhibitors, angiotensin receptor blockers, and calcium channel blockers) compared to older medications (diuretics and alpha- and beta-blockers). These data support the therapeutic recommendations of current hypertension guidelines (Williams et al., 2018).

In summary, this Research Topic provided important results from both basic and clinical science demonstrating all levels of research of this scientific area. We hope that these valuable data will help to implement the knowledge in clinical practice and to improve cardiovascular outcome of patients.
